# Seasonal patterns of neurogenesis in European starlings (*Sturnus vulgaris*) are region‐ and sex‐specific

**DOI:** 10.1111/jne.13455

**Published:** 2024-10-16

**Authors:** Sean D. T. Aitken, Broderick. M. B. Parks, Marjorie Sollows, Colleen A. Barber, Leslie S. Phillmore

**Affiliations:** ^1^ Department of Psychology and Neuroscience Dalhousie University Halifax Nova Scotia Canada; ^2^ Department of Biology Saint Mary's University Halifax Nova Scotia Canada

**Keywords:** neurogenesis, seasonality, songbird, European starling, vocal control system

## Abstract

Songbird vocal behavior, physiology, and brains—including neurogenesis—change between seasons. We examined seasonal differences in neurogenesis in three brain regions associated with vocal production and learning, HVC (letter‐based proper name), robust nucleus of the arcopallium (RA), and Area X, and two brain regions associated with auditory perception, caudomedial nidopallium (NCM) and caudomedial mesopallium (CMM). To do this, we captured wild male and female European starlings (*Sturnus vulgaris*) in spring and fall, collected a blood sample, and minimized time from capture to tissue collection to limit suppressive effects of captivity on neurogenesis. We quantified neurogenesis using doublecortin (DCX) immunohistochemistry, counting new neurons of three DCX cell morphologies (multipolar, fusiform, and round). We found regional differences in types of morphologies expressed, and amount of neurogenesis across regions: NCM had more fusiform cells than all other regions, and RA had more round cells than other regions. Males had more neurogenesis in HVC in fall than in spring, but there was no seasonal difference in neurogenesis in HVC of females, perhaps reflecting sexually dimorphic vocal learning demands related to repertoire size and complexity. Plasma corticosterone was higher in spring than fall and was correlated with testis volume in males, but it was not correlated with another purported measure of stress, heterophil:lymphocyte ratio (HLR), nor with neurogenesis. Our results suggest that the addition of new neurons to specific regions and circuits may serve different functions for males and females, particularly in the context of vocal production, learning, and perceptual demands across seasons.

## 1 INTRODUCTION

Across the annual cycle, songbirds display remarkable levels of change, including in vocal behavior and physiology, particularly between breeding season and nonbreeding season.^1^, ^2^ There is also evidence for seasonal neural plasticity, including changes in volume and neuron number, in some regions of the vocal control system, a series of interconnected nuclei underlying vocal production, perception, and learning.^3^, ^4^, ^5^, ^6^, ^7^ Temperate songbirds provide a distinctive opportunity to study seasonal change in adult neurogenesis—the proliferation, differentiation, migration, and incorporation of new neurons to neural circuits—because neurogenesis remains elevated and extensive after development.^8^, ^9^, ^10^ To date, studies of neurogenesis have tended to focus on one vocal control nucleus, HVC (letter‐based proper name), typically only in males, and generally have not considered seasonal changes, especially in wild‐caught species.^5^, ^11^, ^12^ Here, we examine simultaneously seasonal and sex differences in neurogenesis in both vocal control and auditory perceptual regions in a wild‐caught open‐ended vocal learner, the European starling (*Sturnus vulgaris*).

Results of studies that examine seasonal neurogenesis in songbirds are mixed. In HVC, part of both direct (projecting to the robust nucleus of the arcopallium, RA) and indirect (projecting to RA via basal ganglia nucleus Area X) motor pathways in the vocal control system,^6^, ^13^ neurogenesis is highest in males in breeding condition compared with nonbreeding condition in some species (e.g., Indian weaver birds, *Ploceus philippinus*; red‐headed buntings, *Emberiza bruniceps* ^14^; white‐crowned sparrows, *Zonotrichia leucophrys gambelii* ^15^, ^16^), suggesting the addition of new neurons to HVC during breeding season may support changes to song duration and stereotypy. In other species (e.g., canaries, *Serinus canaria*; red‐winged blackbirds, *Agelaius phoeniceus*; brown‐headed cowbirds, *Molothrus ater*) nonbreeding males have more neurogenesis than breeding males,^12^, ^17^, ^18^ suggesting that neurogenesis in HVC may also facilitate the addition of new vocal elements after breeding. Interestingly, Guigueno et al.^12^ found female cowbirds have the opposite seasonal pattern to males (i.e., more neurogenesis in breeding season than nonbreeding season), suggesting neurogenesis may underlie different processes in male and female birds, and ultimately emphasizing the importance of considering sex differences in seasonal neurogenesis.^5^

There are few studies examining seasonal patterns of neurogenesis in vocal control regions other than HVC. There were no seasonal differences in neurogenesis in Area X in male song sparrows (*Melospiza melodia* ^19^) nor Indian weaver birds,^14^ but there was a difference in male red‐headed buntings.^14^ Many studies report little or no neurogenesis in RA of adult songbirds^20^, ^21^, ^22^ (but see reference 23); the well‐documented seasonal changes in RA volume are purported to be associated with changes in RA cytoarchitecture (e.g., neuron size, spacing, dendritic spine density), rather than addition of new neurons.^7^

Regions associated with perception of acoustic stimuli are also an important consideration, especially in the context of a seasonally changing acoustic landscape for temperate birds. There is extensive evidence that both the caudomedial nidopallium (NCM) and caudomedial mesopallium (CMM) are active while processing auditory information but may be functionally distinct: NCM seems to be important for memory of vocalizations during vocal learning and individual recognition while CMM seems to be important for discrimination of conspecific vocalizations.^24^, ^25^ There is seasonal variation in neuronal activation in NCM and CMM: expression of the immediate‐early gene ZENK is higher in nonbreeding black‐capped chickadees (*Poecile atricapillus*) than breeding chickadees^26^ (but see reference 27). Given that neuronal activation is linked to neurogenesis in HVC^28^ and there is neurogenesis in these auditory perceptual regions,^29^, ^30^, ^31^ it is surprising that studies examining seasonal variation of neurogenesis in auditory perceptual regions are limited. Surbhi et al.,^14^ however, did show that compared with nonbreeding birds, neurogenesis was higher in NCM and CMM of both breeding and prebreeding Indian weaver birds and red‐headed buntings, but this is, to our knowledge, the only study to date which has examined seasonal changes in neurogenesis in these auditory perceptual regions.

In this study, we examined neurogenesis in both vocal control (HVC, RA, Area X) and auditory perceptual regions (NCM, CMM) of male and female European starlings captured from the wild in spring and fall. Both male and female starlings are open‐ended learners,^32^, ^33^, ^34^ adding to their vocal repertoire over their lifetime—making this species an ideal wild‐caught complement to studies of laboratory‐reared canaries, who are also open‐ended learners.^35^, ^36^, ^37^ There is evidence of seasonal neural plasticity in male starlings: wild‐caught males have larger HVC, RA, and Area X by volume in spring than in fall,^38^ a phenomenon partially replicated in starlings housed in the laboratory.^39^ However, there were no differences in neurogenesis in HVC in captive nonbreeding males treated with testosterone (i.e., to induce changes similar to those of birds in breeding condition) compared with nontreated control birds.^11^

Here, we included both males and females in our study design and did not hold birds in captivity longer than 1 h before sacrifice to minimize any potentially inhibitory effect of captive housing on the brain, including on neurogenesis.^40^, ^41^ We quantified labeling of the microtubule‐associated protein, doublecortin (DCX), a well‐used endogenous marker of newly born neurons in birds,^42^ as our measure of neurogenesis. Further, given that corticosterone (CORT), a glucocorticoid hormone associated with the vertebrate stress response^43^ is associated with decreased neurogenesis in HVC and the subventricular zone^44^ and varies seasonally in many temperate songbirds, including starlings,^45^, ^46^ we also quantified baseline CORT in plasma samples collected from our subjects at the time of capture. Blood samples were also used to quantify an alternative indicator of stress and immune function in birds, heterophil:lymphocyte ratio (HLR)^47^, ^48^, ^49^. HLR follows a more protracted timeline than that of plasma CORT^50^ and, therefore, may be a better indicator of baseline stress levels after capture.

## 2 METHOD

All experimental procedures were approved by the Dalhousie University Committee on Laboratory Animals (protocol #17–120) in accordance with the guidelines of the Canadian Council on Animal Care. Birds were captured and handled under a scientific permit issued by the Nova Scotia Department of Natural Resources (Wildlife Division).

### 2.1 Subjects

We captured 16 European starlings (10 males, 6 females) on the campus of Dalhousie University in Halifax, Nova Scotia (44.64° N, 63.59° W) using walk‐in Potter traps baited with Doritos®, Hickory Sticks and/or French fries. We captured breeding birds (*n* = 8; 6 males, 2 females) from May 17 to May 19, 2018 (photoperiod range: 14.9 L:9.1D–15.3 L:8.7D) and nonbreeding birds (*n* = 8, 4 males, 4 females) from October 22 to December 13, 2018 (photoperiod range: 10.7 L:13.3D–8.8 L:15.2D).

### 2.2 Blood, feather, and tissue collection

All birds were captured between 10:00 and 14:30, at least 120 min after local sunrise time to avoid diel variation in circulating CORT; CORT is highest during the inactive nighttime period and lowest during the active daytime period across seasons.^46^, ^51^ Once a bird entered a trap, we immediately extracted the bird to ensure all blood was collected within 3 min of capture, standard practice when quantifying baseline CORT in birds,^52^, ^53^, ^54^ including in starlings^46^ we collected blood into a maximum of six 70 μL heparinized microcapillary tubes per bird by making a small puncture in the left brachial vein with a 26‐gauge needle tip. After collection, we stored sealed tubes on ice until processing in the laboratory. One tube was used for blood smear analysis (described below), and the others were spun in a microhematocrit centrifuge at 16,000 *g* for 10 min; plasma was then aliquoted and stored at −80°C until samples were assayed for CORT (described below).

After blood collection in the field, we collected eight hackle feathers to later determine birds' age (described below). Then, birds were placed in cloth bags and transferred to the laboratory within 1 h of capture. Birds were weighed, euthanized via intraperitoneal injection of xylazine and Euthanyl (0.02 mg/kg; 1:1), and transcardially perfused, first with heparinized phosphate buffered saline (PBS) followed by 4% buffered paraformaldehyde. Brains were harvested and stored in paraformaldehyde for 48 h until uniformly fixed, followed by 24 h in 30% sucrose (in PBS) until saturated. Brains were then flash frozen on pulverized dry ice and stored at −80°C until sectioning.

After perfusion, we removed the left testis from male birds and measured its length and width to the nearest 0.1 mm using a Vernier caliper. These measurements were then used to calculate testis volume using the formula of an ellipsoid:

$$\frac{4}{3}\pi a^{2}b$$

where *a* = width/2 and *b* = length/2. Spermatogenesis in male starlings begins when testes reach a width of 5.5 mm^55^ or volume of 125 mm^3^ ^56^; we therefore considered birds with testes greater than 125 mm^3^ to be in breeding condition. In females, we visually assessed the stage of ovary development using a five‐point ordinal scale originally described for black‐capped chickadees by MacDougall‐Shackleton et al.^57^ and used in European starlings by Stevenson et al.^58^ We considered an ovary stage of 3 (small uniform follicles), 4 (hierarchical follicles) or 5 (large yolky follicles) as indicative of breeding condition (as in reference 59).

### 2.3 CORT assay

We used a commercially available enzyme‐linked immunosorbent assay (ELISA) kit (Enzo Life Sciences; ADI‐900‐097, RRID:AB_2307314) to quantify baseline CORT in plasma samples. This ELISA kit has been used extensively to quantify plasma CORT in songbirds.^60^, ^61^ All plasma samples were diluted to 1:40 according to the small‐volume protocol described by the manufacturer; briefly, 10 μL of steroid displacement reagent (diluted 1:100 in distilled water) was added to 10 μL of raw plasma and then diluted with 380 μL of assay buffer.

All samples were assayed in duplicate; samples with a coefficient of variation greater than 20% (*n* = 2) were excluded from analyses. One sample did not run, and an additional sample read at more than two standard deviations from the mean and was inconsistent with previously published data^46^; both were excluded from analyses. Plasma samples from three birds (all nonbreeding) read below the minimum sensitivity of the assay (prior to adjusting for the dilution factor); these data were adjusted to the minimum sensitivity (27 pg/mL; 1080 pg/mL adjusting to dilution factor) in our statistical analyses. Intra‐assay variation for plasma samples was 7.8%, consistent with the value reported by the manufacturer (8.0%). We did not assess inter‐assay variation as all samples were assayed on one plate; inter‐assay variation is reported by the manufacturer as 13.1%.

### 2.4 Blood Smear Analysis

We prepared blood smears by placing a drop of whole blood from one capillary tube onto a microscope slide and used the edge of another slide to produce a smear. After drying, slides were fixed with 95% methanol and stained using the Hema 3™ procedure (cat no. 22–122911; Fisher) and then coverslipped with Permount (Fisher).

We analyzed blood smears using an Olympus CX23 microscope with oil immersion at ×1000 magnification. Following previous work^50^ we identified the monolayer of the blood smear and worked from the top of the slide down, marking the bottom of one field of view (FOV) to establish the top of the next until reaching the bottom of the smear. Once at the bottom we returned to the top of the smear and to the right, ensuring no FOV overlapped with another. For each FOV, we counted the white blood cells (WBCs) and identified each as either basophil, eosinophil, heterophil, lymphocyte, or monocyte before moving to the next FOV. We estimated the number of red blood cells (RBCs) by counting the number of RBCs on one half of the FOV, switching between sides for each FOV. We repeated this process on each slide until we counted 100 WBCs; we then estimated the number of WBCs per 10,000 RBCs and calculated the heterophil:lymphocyte ratio (HLR).

### 2.5 Aging

We assessed the age of our subjects by measuring total hackle length and length of hackle iridescence (to the nearest 0.1 mm) based on previously established protocols.^62^, ^63^ Hackles were examined using a dissecting microscope. Hackle length was measured from the hackle tip to the base of the main shaft of the feather; length of hackle iridescence was measured from the tip to where no iridescence was present. Birds were assigned to either second year (SY; captured in its second calendar year) or after second year (ASY; an adult in at least its third calendar year^64^) based on length of hackle iridescence according to the method established by Kessel.^63^ Males were considered SY if iridescence length was <11.0 mm, and ASY if it was >11.0 mm. Females were considered SY if iridescence length was <6.5 mm, and ASY if it was >6.5 mm. Of all birds captured, 14/16 (8 males, 6 females) were aged as ASY; 2/16 (both males captured in fall) were aged as SY.

### 2.6 Immunohistochemistry

Brains were sectioned in the coronal plane using a cryostat (−17°C; 12° blade angle) at a thickness of 50 μm into three sets; every third section (150 μm apart) was used for DCX immunohistochemistry. Sections were then stored in cryoprotectant (30% sucrose, 30% ethylene glycol, 1% polyvinylpyrrolidone in PBS) at −20°C until immunohistochemistry was performed. Prior to immunohistochemistry, birds were assigned a random three‐digit identification number such that all subsequent tissue processing, microscopy, and quantification could be completed blind to capture season and sex.

First, sections were removed from the freezer and washed five times (PBS) to remove cryoprotectant. Unless otherwise indicated, all washes were 5 min with agitation, and all steps were carried out at room temperature. Tissue was then incubated in 0.5% hydrogen peroxide for 30 min, then washed three times (PBS) and incubated in 10% normal goat serum (Vector, S‐1000) in 0.3% Triton‐X in PBS (PBS/T) for 1 h. Primary antibody (rabbit anti‐DCX IgG, Abcam, ab18723, RRID:AB_732011) was prepared in 0.3% PBS/T at a concentration of 1:2000; tissue was incubated for 24 h at 4°C. The primary anti‐DCX antibody used for immunohistochemistry targets amino acid residues 300 to the C‐terminus of human DCX (UniProt: O43602), a sequence highly conserved between birds and mammals.^20^, ^21^, ^65^ The specific immunogen sequence used to raise the antibody shares 100% identity (i.e., complete overlap) with the orthologous sequence for DCX in European starlings (RefSeq: XP_014732643.1). This antibody has been characterized for use in birds^66^ and has been used extensively to quantify DCX immunoreactivity (DCX‐ir) in a variety of songbird species (e.g., canaries^67^, ^68^, ^69^; zebra finches, *Taeniopygia castanotis* ^70^). Tissue stained using this antibody in this study yielded a staining distribution consistent with previously published examples of DCX‐ir in the songbird telencephalon using different anti‐DCX antibodies.^12^, ^23^, ^71^, ^72^ Omission of the primary antibody from the staining protocol produced no staining.

After incubation in primary antibody, tissue was washed twice (0.1% PBS/T) and then incubated in biotinylated secondary antibody (goat‐anti‐rabbit IgG (H + L), Vector, BA‐1000, RRID:AB_2313606) at 1:500 in 0.3% PBS/T for 1 h. The tissue was then washed three times (0.1% PBS/T) before being incubated in avidin‐biotin horseradish‐peroxidase complex (VECTASTAIN Elite ABC‐HRP Kit, Vector, PK‐6100, RRID:AB_2336819) at a concentration of 1:200 in 0.3% PBS/T for 1 h. Tissue was washed twice (0.1% PBS/T) then visualized using SIGMAFAST™ DAB (Sigma‐Aldrich) for 90 s, then washed five times (PBS) before being float mounted onto gelatin‐coated slides, serially dehydrated in increasing concentrations of ethanol, cleared with NeoClear (Harleco), and coverslipped with Permount.

### 2.7 Microscopy and quantification of DCX‐ir

**FIGURE 1:**
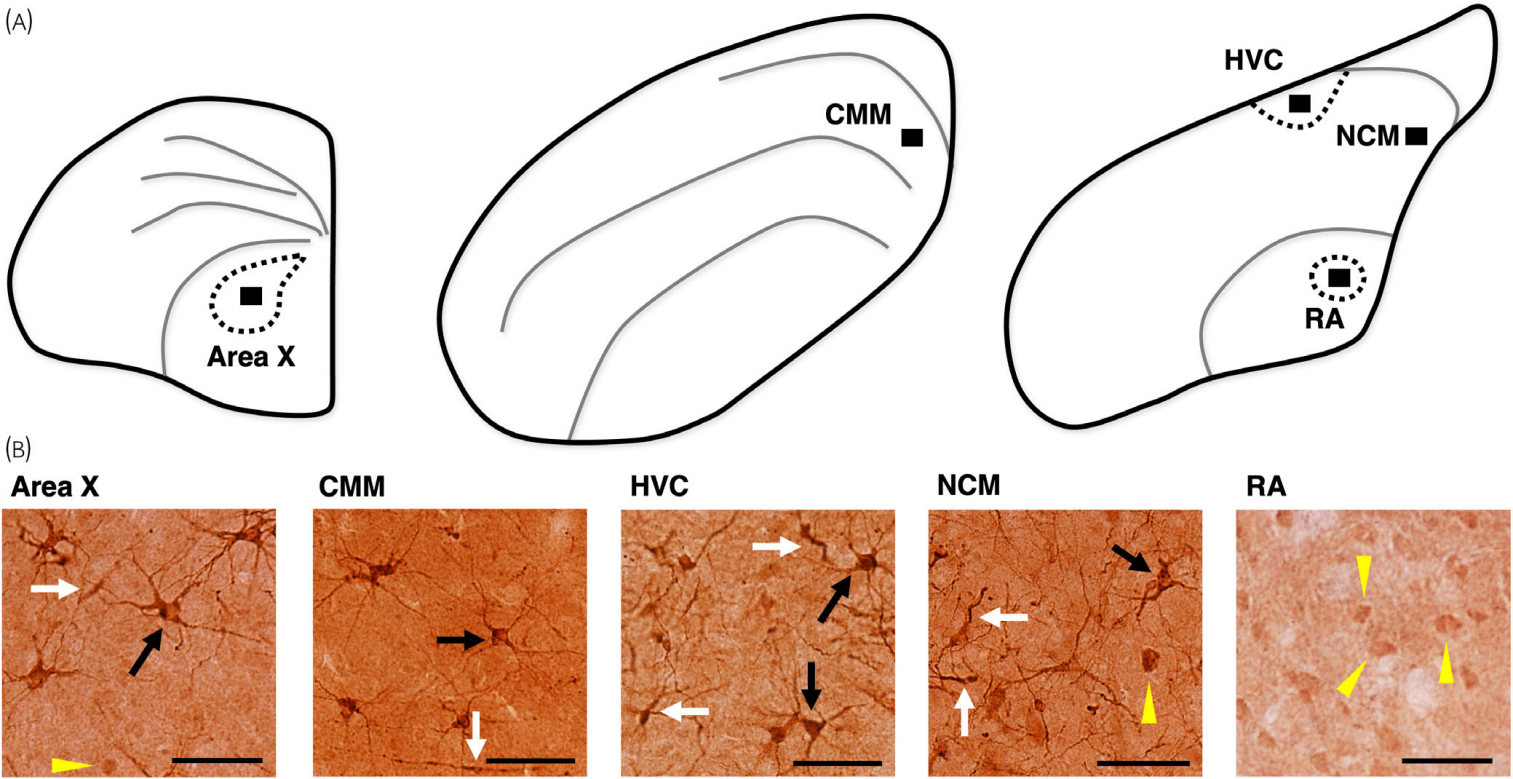
(A) Line drawings of one hemisphere of the European starling telencephalon (coronal) across three representative planes of section (rostral‐caudal; from left to right), showing each of the five regions of interest (from rostral to caudal: Area X, caudomedial mesopallium [CMM], HVC [letter‐based proper name], NCM [caudomedial nidopallium], robust nucleus of the arcopallium [RA]) in this study. (B) Representative images of doublecortin‐immunoreactive (DCX‐ir) tissue (visualized with SIGMAFAST™ DAB) from each brain region of interest (×400 magnification; scale bars = 50 μm). Black arrows indicate examples of DCX‐ir multipolar cells, white arrows indicate examples of DCX‐ir fusiform cells, yellow arrowheads indicate examples of DCX‐ir round cells.

To quantify DCX‐ir in our brain regions of interest (ROIs), we imaged slides under brightfield illumination using an Olympus DP80 camera paired to an Olympus BX51 microscope at ×400 magnification. Images were captured using Olympus cellSens Dimension software (version 1.14). We quantified DCX‐ir in five brain regions: two auditory perceptual regions (NCM, CMM; Figure 1) and three vocal control nuclei (HVC, RA, and Area X; Figure 1) using the stereotaxic atlas of the zebra finch brain^73^ for reference; we also relied on previously published reference images of NCM and CMM in the coronal plane^74^ to additionally confirm the location of our images. We captured up to three images (per hemisphere) in successive sections for each of the five brain regions. Within each image (*n* = 344 total; dimensions = 220 × 165 μm; area = 0.0363 mm^2^), we exhaustively counted three types of DCX‐ir cells based on morphology: multipolar, fusiform, and round. Figure 1 shows representative images of DCX‐ir from each ROI (rostral to caudal); examples of each DCX‐ir cell type we quantified are also indicated within the images. Multipolar cells exhibited distinct cell bodies with multiple processes emanating from them; fusiform cells had elongated cell bodies with spindle‐shaped processes (both categories described previously by references 12, 20, 21, 71, 75; among others). We also counted a separate morphology (hereafter “round”) with a distinct cell body but lacking observable processes; these cells tended to be (but were not always) less densely stained than multipolar or fusiform cells. This DCX‐ir round morphology has described and counted by several previous studies with varying nomenclature, including “hippocampal spherical” in starlings^71^ and “spherical” in corvids.^76^ This morphology has also been described as “weakly stained round with few processes” in canaries^20^, ^21^ and cowbirds,^75^ but was not counted separately from the other DCX‐ir cell types and/or was counted together with DCX‐ir multipolar cells.

Images were imported into ImageJ^77^ where we used the ‘Threshold’ function to quantify the area of the image that was DCX‐ir (“%DCX‐ir coverage”) after the image was converted to 32‐bit grayscale. Thresholding was confirmed by eye to ensure background immunoreactivity was excluded (as in reference 72). While some previous studies^23^, ^41^, ^72^ have opted to use %DCX‐ir coverage exclusively to quantify DCX‐ir in the songbird brain; here we use it as a complementary measure to our cell count data (as in references 12, 22, 78), as coverage allows us to account for any staining from processes or arborization not obviously connected to a cell body, as well as ambiguous or uncategorizable cell types.

### 2.8 Statistical analyses

All analyses were conducted using jamovi (version 2.3.21^79^). We used the GAMLj (General Analyses for Linear Models in jamovi; version 2.6.6^80^) module to probe for seasonal and sex differences in our dependent measures. We used different models based on the type of data (e.g., continuous vs. counts) being analyzed; more information on each model is described in the appropriate results section. Post hoc tests consisted of multiple pairwise comparisons; all *p*‐values derived from pairwise comparisons were adjusted using the Bonferroni correction. We probed interaction effects using simple effects tests. Significance was set at *α* = .05.

We also conducted correlational analyses (using Pearson's *r*) to assess relationships among physiological measures (testis volume in males, plasma CORT, HLR) and neurobiological measures (DCX‐ir cell counts and %DCX‐ir coverage). We used raw data for correlations within our DCX variables (counts and coverage) and averages of counts and coverage (averaged across hemisphere and tissue section for each individual bird) for correlations with physiological variables. Data were visualized using RStudio (version 2023.12.1.402^81^); individual data points are represented for physiological data and all correlations. Estimated marginal means and bootstrapped 95% confidence intervals (CIs) are presented for all neurogenesis measures, as each individual bird contributed multiple data points in our statistical analyses.

## 3 RESULTS

### 3.1 Physiology

#### 3.1.1 Gonads

**FIGURE 2:**
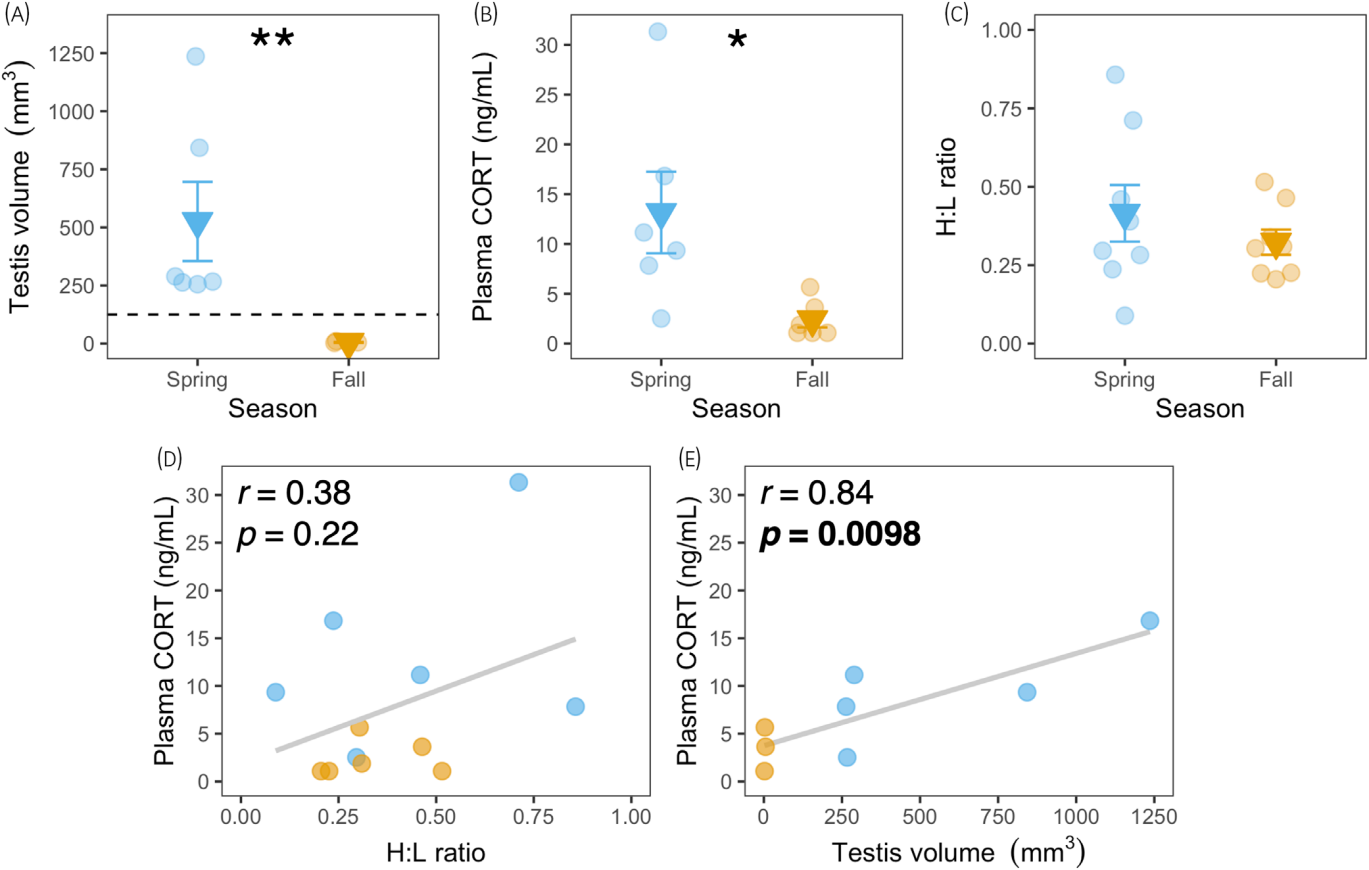
Seasonal differences in physiological measures in European starlings. (A) shows testis volume in males; dashed line indicates 125 mm^3^ threshold for breeding condition described by Dawson.^56^ (B) shows plasma corticosterone (CORT); (C) shows heterophil:lymphocyte ratio (HLR). CORT was not significantly correlated with HLR (D), but was significantly correlated with testis volume in males (E). Dots are individual data (A−E); darker triangles ± error bars (A–C) indicate group means ± SEs. Asterisk(s) indicate(s) a seasonal difference at *p* < .05 (*) and *p* < .001 (**) when analyzing natural‐log transformed data (A, B), not shown for ease of visualization. Solid lines (D, E) are regression lines; significant correlations (as determined by Pearson's *r*) at *p* < .05 are indicated by bolded text (E).

We analyzed gonad data from males and females separately. Figure 2A shows seasonal differences in testis volume in males. All six males captured in spring had large testes above the diametric and volumetric thresholds for breeding condition^55^, ^56^ (mean testis volume ± SE = 530.5 ± 173.1 mm^3^); the four males captured in fall condition had small testes below these thresholds (mean volume ± SE = 5.25 ± 1.89 mm^3^). An independent samples *t*‐test revealed a significant difference in testis volume (natural‐log transformed) between our groups (*t*_8_ = 10.10; *p* < .0001, Cohen's *d* = 6.52).

Of the two females captured in spring, one had hierarchical ovaries (stage = 4) and one had granular ovaries (stage = 2); all females captured in fall had granular ovaries (Figure S1). Given unequal sample sizes and the ordinal nature of the scale used to assess stage of ovary development, we assessed seasonal differences using a nonparametric Mann–Whitney *U* test (as in references 59, 82) and found no effect of season (*U* = 2.00, *p* = .29).

#### 3.1.2 Plasma CORT and HLR

Figure 2B shows raw baseline plasma CORT levels for birds captured in spring and fall. We analyzed CORT data using a general linear model (GLM) with CORT (natural‐log‐transformed) as the dependent variable and season and sex as fixed factors. We found a main effect of season on plasma CORT (*F*_1,8_ = 20.8, *p* = .0018, *ω* ^2^ = .61); birds captured in spring had higher plasma CORT than those captured in fall (*p* = .0018). There were no main effects of sex (*F*_1,8_ = 0.39, *p* = .55, *ω* ^2^ < 0), nor a season × sex interaction (*F*_1,8_ = 5.23, *p* = .052, *ω* ^2^ = .13).

Figure 2C shows HLR for birds captured in spring and fall; we tested for seasonal and sex differences in HLR using a GLM with HLR as the dependent variable and season and sex as fixed factors. We found no seasonal differences (*F*_1,12_ = 1.12, *p* = .31, *ω* ^2^ = .01), sex differences (*F*_1,12_ = 0.48, *p* = .50, ω^2^ < 0), nor a season × sex interaction (*F*_1,12_ = 0.07, *p* = .79, *ω* ^2^ < 0) in HLR.

Plasma CORT was not significantly correlated with HLR (*r* = .38, *p* = .22; Figure 2D) but was significantly correlated with testis volume in males (*r* = .84, *p* = .0098; Figure 2E).

### 3.2 Neurogenesis

**FIGURE 3:**
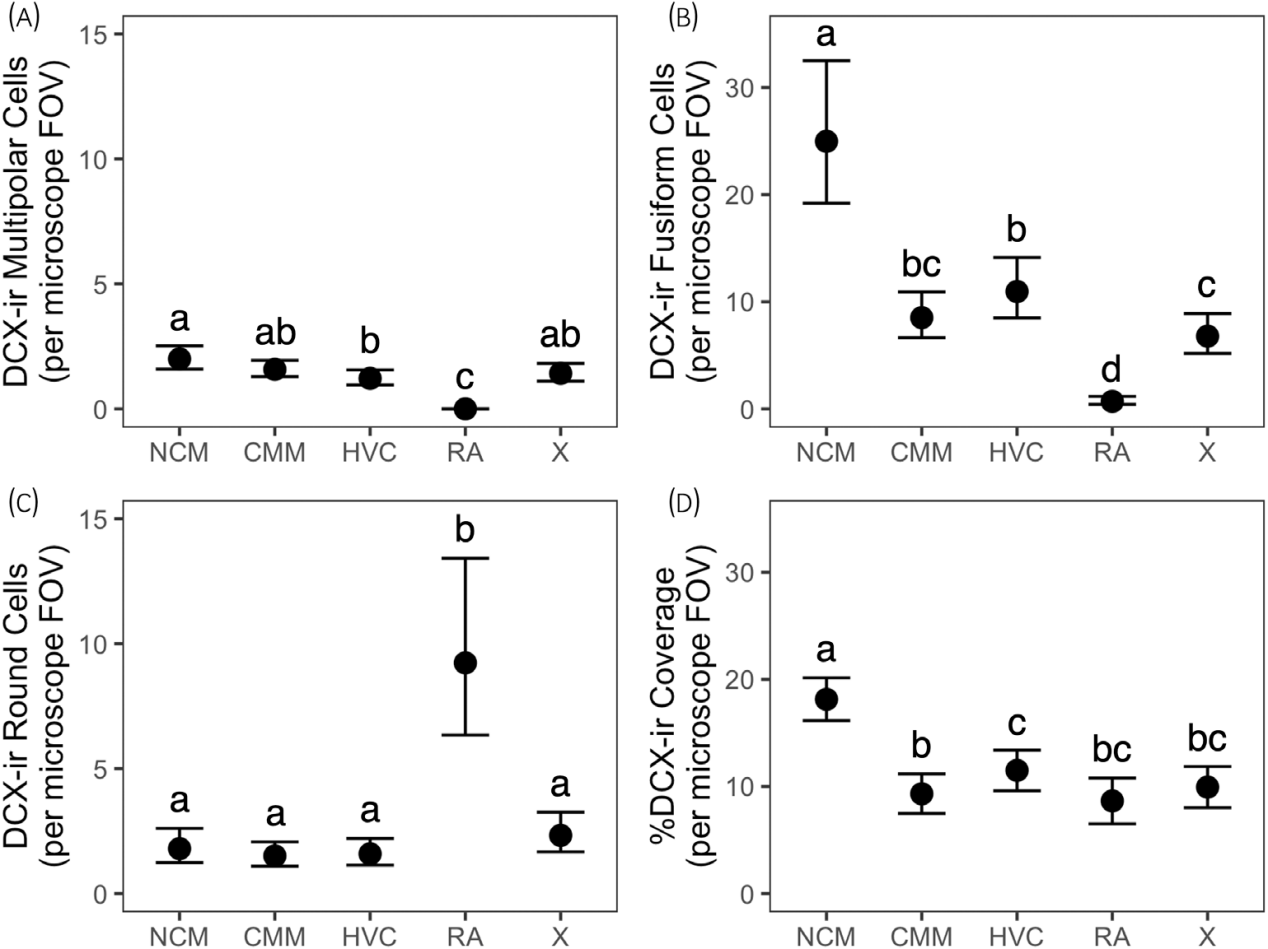
Regional differences in counts of doublecortin‐immunoreactive (DCX‐ir) multipolar cells (A), fusiform cells (B), round cells (C), and in %DCX‐ir coverage (D) in European starlings. Data presented are estimated marginal means ± bootstrapped 95% confidence intervals (CIs) generated from generalized linear mixed models (A–C) and a general linear mixed model (D). Regions not sharing a letter indicate a significant regional difference at Bonferroni‐adjusted *p* < .05. CMM, caudomedial mesopallium; FOV, field of view; HVC, HVC (letter‐based proper name); NCM, caudomedial nidopallium; RA, robust nucleus of the arcopallium; X, Area X.

**FIGURE 4:**
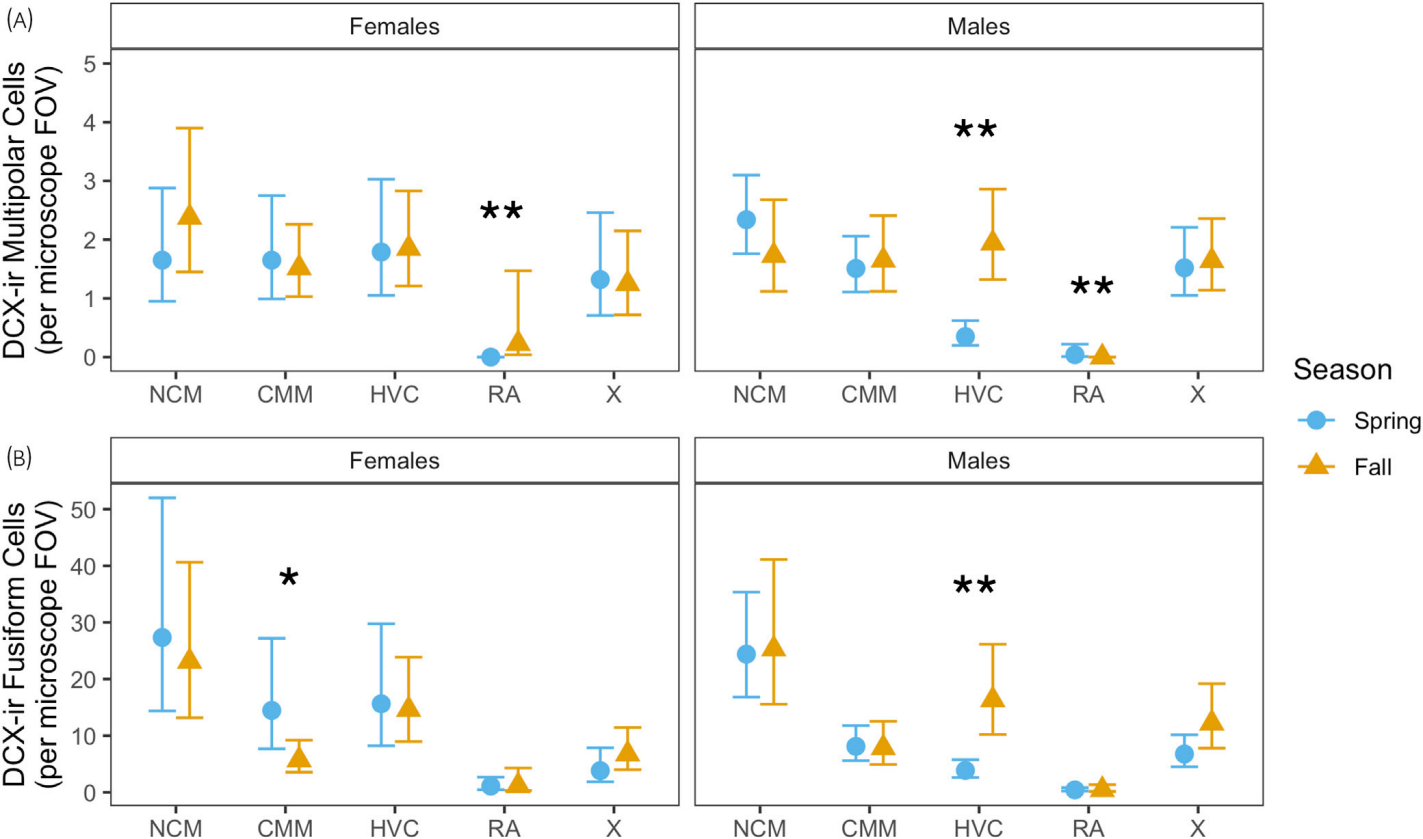
Region‐specific seasonal differences in counts of doublecortin‐immunoreactive (DCX‐ir) multipolar cells (A) and DCX‐ir fusiform cells (B) in female (left) and male (right) European starlings. Blue circles indicate data from birds captured in spring; orange triangles indicate data from birds captured in fall. Data presented are estimated marginal means ± bootstrapped 95% confidence intervals (CIs) generated from generalized linear mixed models. Asterisk(s) indicate(s) significant region × season × sex interaction effects at *p* < .05 (*) and *p* < .001 (**). CMM, caudomedial mesopallium; FOV, field of view; HVC, HVC (letter‐based proper name); NCM, caudomedial nidopallium; RA, robust nucleus of the arcopallium; X, Area X.

Figure 3 shows mean counts ± bootstrapped 95% CIs of multipolar (A), fusiform (B) and round (C) DCX‐ir cell types and mean  ± bootstrapped 95% CIs of %DCX‐ir coverage (D) in all five regions: NCM, CMM, HVC, RA, and X. Figure 4 shows mean counts ± bootstrapped 95% CIs of multipolar (A) and fusiform (B) DCX‐ir cell types for spring (blue circles) and fall (orange triangles) birds, separated by sex. All count data were analyzed using generalized linear mixed models (GLMMs). We examined regional, seasonal, and sex differences in DCX‐ir cell counts for each DCX‐ir cell type (multipolar, fusiform, round) separately. We originally fit raw count data to a Poisson distribution, but across all models cell count data were extremely overdispersed. We therefore opted to fit count data to a negative binomial distribution (log link function); this resolved the issue of overdispersion.

Each GLMM included brain region, season, and sex as fixed factors and individual (bird ID) was included as the random factor. We also checked for hemispheric differences in our ROIs using a separate GLMM with region and hemisphere as fixed factors and bird ID as the random factor but we found no main effects of hemisphere, nor any region × hemisphere interaction effects in DCX‐ir multipolar, fusiform, or round cell counts. We therefore did not include hemisphere as a fixed factor in our final models. Omitting hemisphere also improved the fit (i.e., decreased the Akaike Information Criterion) of each model.

#### 3.2.1 DCX counts—multipolar cells

There was a main effect of brain region (*χ* ^2^_4_ = 147.9, *p* < .0001) and season (*χ* ^2^_1_ = 4.67, *p* = .031), but not sex (*χ* ^2^_1_ = 0.08, *p* = .78) on DCX‐ir multipolar cells. Analysis of the main effect of brain region showed RA had significantly fewer multipolar cells than all other regions (all *p*s < .0001). NCM had significantly more multipolar cells than HVC (*z* = 3.23, *p* = .012; Figure 3A). Analysis of the main effect of season showed birds captured in fall had significantly more multipolar cells than birds captured in spring (*z* = −2.16, *p* = .031).

All two‐way interaction effects were significant (all *p*s <.05), as was the three‐way region × season × sex interaction (*χ* ^2^_4_ = 215.7, *p* < .0001). We probed the three‐way interaction further using simple effects, which revealed that male starlings captured in fall had significantly more multipolar cells in HVC than males captured in spring (*χ* ^2^_1_ = 23.8, *p* < .0001), but there was no seasonal difference in HVC in females. Both male and female starlings also had significant seasonal differences in DCX‐ir multipolar cells in RA (both *p*s < .0001; Figure 4A); however, this effect is likely because we observed no multipolar cells in RA images collected from spring females nor fall males, and very few in other groups.

#### 3.2.2 DCX counts—fusiform cells

There was a main effect of brain region (*χ* ^2^_4_ = 307.4, *p* < .0001), but not season (*χ* ^2^_1_ = 0.38, *p* = .54) nor sex (*χ* ^2^_1_ = 0.90, *p* = .34) on DCX‐ir fusiform cells. Multiple pairwise comparisons revealed NCM had significantly more fusiform cells than all other brain regions (all *p*s < .0001), RA had significantly fewer fusiform cells than all other brain regions (all *p*s < .0001), and HVC had significantly more fusiform cells than Area X (*p* < .001; Figure 3B).

We found significant region × season (*χ* ^2^_4_ = 48.6, *p* < .0001) and region × sex (*χ* ^2^_4_ = 36.7, *p* < .0001) interactions but not a season × sex interaction (*χ* ^2^_1_ = 1.12, *p* = .29). The three‐way region × season × sex interaction was also significant (*χ* ^2^_4_ = 17.1, *p* = .018). Further probing this three‐way interaction using simple effects tests revealed one region‐specific seasonal difference for females and one for males: female starlings captured in spring had significantly more fusiform cells in CMM than female starlings captured in fall (*χ* ^2^_1_ = 5.26, *p* = .022; Figure 4B left panel), and male starlings captured in fall had significantly more fusiform cells in HVC than males captured in spring (*χ* ^2^_1_ = 21.1, *p* < .0001; Figure 4B right panel).

#### 3.2.3 DCX counts—round cells

There was a main effect of brain region (*χ* ^2^_4_ = 307.4, *p* < .0001), but not season (*χ* ^2^_1_ = 3.77, *p* = .052) nor sex (*χ* ^2^_1_ = 1.21, *p* = .27) on DCX‐ir round cells. Analyzing the main effect of season revealed RA had significantly more round cells than all other brain regions (all *p*s < .0001; Figure 3C).

The only significant two‐way or three‐way interaction effect was the season × sex interaction (*χ* ^2^_1_ = 5.37, *p* = .020); further probing this interaction with simple effects tests revealed males captured in spring had significantly more round cells than males captured in fall (*χ* ^2^_1_ = 12.93, *p* = .0003), but there was no seasonal difference in females (*χ* ^2^_1_ = 0.07, *p* = .79; Figure S2).

#### 3.2.4 %DCX‐ir coverage

We analyzed %DCX‐ir coverage with a general linear mixed model with %DCX‐ir coverage as the dependent variable, brain region, season and sex as fixed factors, and bird ID as a random factor. As with count data, we omitted hemisphere as a factor in our model as we found no significant hemispheric differences in any of our regions of interest.

We found a main effect of brain region (*F*_4,312.6_ = 43.5, *p* < .001), but not season (*F*_1,12.7_ = 2.26, *p* = .16) nor sex (*F*_1,12.7_ = 0.55, *p* = .47) on %DCX‐ir coverage. Pairwise comparisons revealed NCM had significantly more %DCX‐ir coverage compared with all other brain regions (all *p*s < .0001) and HVC had a significantly more %DCX‐ir coverage than CMM (*t*_312.1_ = −3.24, *p* = .013) and RA (*t*_312.1_ = 3.29, *p* = .011; Figure 3D).

There were significant region × season (*F*_4,312.6_ = 6.06, *p* = .0001) and region × sex interaction effects (*F*_4,312.6_ = 9.01, *p* < .0001), but no season × sex interaction (*F*_1,12.7_ = 0.85, *p* = .37). There was also no three‐way region × season × sex interaction effect (*F*_4,312.6_ = 1.90, *p* = .11). Analyzing the region × season interaction effect with simple effects revealed significantly more %DCX‐ir coverage in fall birds in HVC (*F*_1,20.7_ = 7.70, *p* = .012) and RA (*F*_1,35.4_ = 4.84, *p* = .034; Figure S3) but not in any other regions. Analyzing the region × sex interaction revealed a sex difference in %DCX‐ir coverage in Area X, where males had higher %DCX‐ir coverage than females (*F*_1,21.9_ = 6.73, *p* = .017; Figure S4).

### 3.3 Correlations

Correlation analyses among physiological and neurobiological measures revealed only two significant correlations: HLR was negatively correlated with DCX‐ir multipolar cells in NCM (Pearson's *r* = −.61, *p* = .025; Figure S5), and testis volume in males was positively correlated with round cells in HVC (*r* = .70, *p* = .036; Figure S6), although this correlation did not persist when testis data from one outlier (one male bird captured in spring) was excluded from analyses (*r* = .04, *p* = .921).

**FIGURE 5:**
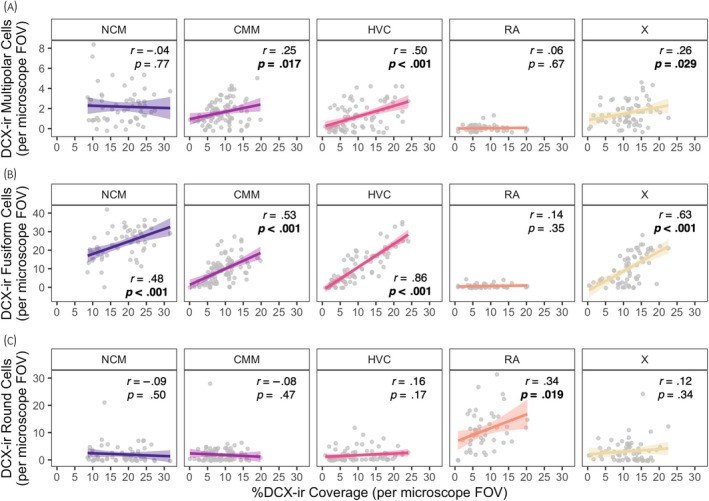
Correlations between % doublecortin‐immunoreactive (DCX‐ir) coverage and DCX‐ir multipolar (A), fusiform (B), and round (C) cell counts, separated by brain region. Grey dots are individual count and coverage data (1 dot = 1 image); solid lines ± bands (in colour) indicate regression lines ± 95% confidence intervals (CIs). Significant correlations (as determined by Pearson's *r*) at *p* < .05 are indicated by bold text. CMM, caudomedial mesopallium; FOV, field of view; HVC, HVC (letter‐based proper name); NCM, caudomedial nidopallium; RA, robust nucleus of the arcopallium; X, Area X.

Figure 5 shows correlations among individual image %DCX‐ir coverage and DCX‐ir cell count within that image for each cell morphology (multipolar, Figure 5A; fusiform, Figure 5B; round, Figure 5C) separated by brain region. Correlation analyses showed %DCX‐ir coverage was positively correlated with fusiform cell counts in all regions (*r*s = .48–.63; all *p*s ≤.0001) except RA (*r* = .14, *p* = .35; Figure 5B). Coverage was correlated with multipolar cells in CMM (*r* = .25, *p* = .017), HVC (*r* = .50, *p* < .0001), and Area X (*r* = .26, *p* = .029), but not in NCM or RA (*p*s >.05; Figure 5A). Coverage was correlated with round cell counts only in RA (*r* = .34, *p* = .019) but in no other region (*p*s >.05; Figure 5C).

## 4 DISCUSSION

In this study, we examined whether neurogenesis varied seasonally in male and female starlings captured in spring and fall. We quantified neurogenesis using DCX immunohistochemistry in three nuclei of the vocal control system (HVC, RA, Area X) and two regions important for auditory perception and processing (NCM and CMM). We minimized the time between capture and sacrifice to mitigate any suppressive effects of captivity on neurogenesis.^40^, ^41^ Overall, rates of neurogenesis and patterns of DCX‐ir cell morphologies varied across our regions of interest. Seasonal differences in neurogenesis were restricted to specific brain regions in males and largely absent from the few females we examined, suggesting that the addition of new neurons to specific regions and circuits may serve different functions for males and females. However, the small number of females captured in spring (*n* = 2) limits our ability to draw strong conclusions from analyses with this group.

### 4.1 Neurogenesis varies across vocal control and auditory perceptual regions

We used the endogenous protein DCX as our measure of neurogenesis because our minimal‐captivity protocol did not allow the time required for administration and incorporation of exogenous markers (e.g., 5‐bromo‐2′‐deoxyuridine, BrdU^83^). In birds, DCX is a valid and reliable marker of neurogenesis^42^ (but see reference 65) and has been used extensively to quantify adult neurogenesis in the songbird telencephalon, including in starlings.^71^, ^72^ Another advantage of DCX is that it is expressed throughout the neuron, as opposed to other markers (like BrdU) that simply visualize the cell nucleus; this allows for the examination of several distinct morphologies of immature neurons.^83^

Here, we quantified three distinct DCX‐ir cell morphologies as well as %DCX‐ir coverage, a measure of overall DCX‐ir. Although nomenclature of these DCX‐ir cell morphologies varies across papers, two types are consistently identified and quantified: multipolar, purported to be cells that have arrived at a final destination and are integrating into its final circuits, and fusiform, purported to be migrating young neurons.^20^, ^42^, ^83^ Overall, there were more fusiform cells than any other DCX‐ir cell type (Figure 3); this finding is not unexpected; DCX is expressed in neurons for up to approximately a month postmitosis,^42^, ^83^, ^84^ and at least in songbirds, for most of these ca. 30 days, it appears DCX‐ir cells adopt the fusiform morphology.^20^

NCM had more multipolar cells than HVC, and more fusiform cells and higher %DCX‐ir coverage than all other regions (Figure 3). Because we could neither quantify vocalizing nor assess what birds were hearing immediately prior to capture, we cannot explicitly link neurogenesis in NCM to an individual's behavior or acoustic environment. However, in general terms, relatively high levels of neurogenesis of both migrating (i.e., fusiform) and incorporating (i.e., multipolar) cells in NCM is perhaps indicative of the importance of auditory perception throughout the year. Neurogenesis in the hippocampus supports spatial memory demand;^85^ there would be corresponding demand on NCM for formation and storage of auditory memories related to individual recognition and song learning.^86^, ^87^ NCM would be engaged throughout the year, in recognition of mates and offspring in spring and early summer, of flock members at other times of year, and when adding to open‐ended vocal repertoire.^88^, ^89^, ^90^ So, it is possible that neurogenesis in NCM is higher than other regions because memory demand is not only higher, but high year‐round.

We also quantified a third morphology of DCX‐ir cells which we called round cells. Surprisingly, we found that RA had more DCX‐ir round cells than any other region (HVC, X, NCM and CMM). It is generally understood that newborn neurons are not recruited to RA in adult songbirds,^7^ including in starlings.^11^ It is therefore intriguing that we document the presence of DCX‐ir round cells in this nucleus, in contrast to previous studies using DCX‐ir.^20^, ^21^, ^22^ These previous studies typically only quantified multipolar and fusiform DCX‐ir cells—we also report few or no DCX‐ir multipolar and fusiform cells in RA. Interestingly, a recent study by Diez et al.^23^ report DCX‐ir in RA (measured via %DCX‐ir coverage) in adult zebra finches (100–110 days post‐hatch). Inspecting the example images in that study (e.g., figure 8 in reference 23) appears to show DCX‐ir cell types that we would label here as DCX‐ir round cells (Figure 1). Furthermore, and seemingly in corroboration with Diez et al.,^23^ we found a significant correlation between %DCX‐ir coverage and DCX‐ir round cell counts (but not multipolar and fusiform counts), only in RA (Figure 5C).

DCX‐ir cells that resemble what we call round cells have been observed and described in the vocal control system of canaries (e.g., “weakly stained without processes”^21^) and observed and quantified in the hippocampus of starlings (“hippocampal spherical”^71^) and corvids (“spherical”^76^). Whether the DCX‐ir round cells documented here in RA are functionally similar to those observed in hippocampus is not known. While we cannot yet ascribe a particular cell age or function to this round morphology, they appear to make up a considerable proportion of the DCX‐ir cells we and others observe in specific areas of the songbird forebrain (e.g., RA, this study; hippocampus^71^, ^75^, ^76^).

Some suggest that the presence of this round DCX‐ir cell morphology, and even DCX‐ir generally, is not necessarily indicative of neurogenesis per se, but rather cellular plasticity more broadly.^20^, ^21^, ^65^ Indeed, in mammals, DCX is expressed in mature neurons and is related to structural plasticity and cytoskeletal remodeling.^84^, ^91^ We must therefore consider that this is perhaps also true in birds—that is, in some cells, DCX indicates something other than neurogenesis^65^ (but see reference 42). This could explain why we and Diez et al.^23^ found DCX‐ir labeling (particularly of the round morphology) in RA, while other papers (using alternative labels of neurogenesis) have not. However, we argue that the abundance of DCX labeling (whether indicating neurogenesis or other neuronal processes), and particularly the abundance of the DCX‐ir round morphology, as well as their variation with experimental manipulation (e.g., dietary supplementation, exercise,^71^; light exposure,^76^) and possibly even physiology (e.g., correlation with testis volume, Figure S6) warrants reporting and further study to correctly interpret the neurogenic and non‐neurogenic roles of DCX in the songbird brain.

### 4.2 Sex and seasonal differences in vocal control and auditory perceptual regions

Testis volume of all male starlings we captured in spring were significantly larger than testis volume of males captured in fall (Figure 2A) and exceeded the diametric and volumetric thresholds for spermatogenesis,^55^, ^56^ indicating they were in breeding and nonbreeding condition, respectively. Ovaries of females captured in fall were consistent with being in nonbreeding condition (all scores <3); however, of the two females we captured in spring, only one had ovaries indicative of breeding condition (Figure S1). Female songbirds require social and environmental cues in addition to sufficiently long photoperiods to achieve maximum photostimulation and ovary development, in contrast to males whose gonads can fully recrudesce in response to photoperiod alone^5^; it is, therefore, possible this female had begun the transition to breeding condition as it was captured in the months where starlings become photostimulated.^92^, ^93^, ^94^

In vocal control nucleus HVC, there were more DCX‐ir multipolar cells in fall than in spring in males, an effect also observed in red‐winged blackbirds and brown‐headed cowbirds.^12^ In canaries, there is neuronal stability in HVC when males are producing accurate and consistent singing during mating season; this stability breaks down after breeding season when song, and the brain, becomes more plastic.^95^ In other words, increased neurogenesis during the nonbreeding season may support the addition of new song elements to their repertoires. Less recruitment of new neurons to HVC during breeding season is believed to facilitate increased song frequency and stereotypy^96^, ^97^, ^98^ (but see reference 15). It is possible that this also occurs in starlings, an open‐ended learner, and decrystallization during the nonbreeding period leads to increased demand for new neurons in HVC, but a causal relationship between plastic song in fall and the incorporation of new neurons has not yet been demonstrated.^99^ As with multipolar cells, males had more DCX‐ir fusiform cells in HVC in fall than winter. This is consistent with an experiment by Hall and MacDougall‐Shackleton,^72^ who found that male starlings treated with the androgen 5α‐dihydrotestosterone had fewer fusiform cells in HVC than males treated with the antiandrogen flutamide. This supports the idea posited above (and by others) that the recruitment of new neurons to HVC in males supports repertoire modification and expansion—not just with multipolar cells that are incorporated into circuits but also with active recruitment of migrating fusiform cells.

While we note that our method of analyzing DCX‐ir cell counts generates more statistical power from individual data than is possible with physiological data, we also recognize this does not overcome all obstacles associated small group sample sizes, especially our spring female group. Since it is not possible to capture and include additional females without introducing additional confounds (e.g., capture year, reproductive condition, potential batch effects associated with tissue processing), we minimize our subsequent discussion about seasonal differences in females. For example, the lack of seasonal differences in neurogenesis in HVC in females may be entirely due to our small sample and a resulting inability to detect them. However, we speculate that the seasonally‐stable patterns of neurogenesis in HVC in females may correspond to seasonally‐stable female vocal behavior. First, female songs are generally shorter and less complex than those of males^100^ and therefore may not require the same intensity of seasonal change in rates of incorporating DCX‐ir multipolar cells into preexisting neural circuits as do males. Furthermore, female starlings have highly variable songs,^100^ sing throughout the year, including during the nonbreeding season to maintain social cohesion (called ‘gregarious’ song^101^), and discriminate between male‐conspecific songs during breeding season^102^; these changing demands may require consistent (and seasonally unchanging) recruitment of new neurons to HVC. Guigueno et al.^12^ found no seasonal differences in HVC of female blackbirds and cowbirds; we would need more data to determine if this lack of seasonal difference also extends to starlings.

In CMM, a key perceptual region linked to acoustic discrimination^103^, ^104^ we also observed a sex‐specific seasonal pattern of neurogenesis, but opposite to the pattern observed in HVC: there were more fusiform cells in CMM in females captured in spring than in fall, but no difference in males. To our knowledge, this is only the second study (after Surbhi et al.^14^) to examine seasonal changes in neurogenesis within the songbird auditory system. We know relatively little about adult neurogenesis in auditory perceptual regions compared with the vocal control system, and within subregions of the auditory system, we know less about CMM than NCM. Neurogenesis in NCM appears to be modulated by social context^10^; adult zebra finches housed in social groups show an increase in neuronal recruitment in NCM relative to pair‐housed and isolate birds.^105^ Similarly, deafening zebra finches decreases the number of new neurons in NCM^30^; together, these suggest that both acoustic environment and auditory experience modulate neuronal recruitment and survival in this structure.^10^ The function of CMM seems to related to perception of song components and learned stimuli.^25^, ^106^, ^107^ Lesions to CMM prevent female zebra finches from discriminating between conspecific and heterospecific songs^108^ and in female canaries, CMM is more responsive (as measured by *ZENK* mRNA) to complex song syllables compared with less‐complex syllables, suggesting CMM is critical for integrating vocal signals of different quality.^109^ Further, female starlings exposed to long song bouts show significantly more activation (as measured by ZENK‐ir) in CMM and NCM compared with short song bouts after repeated trials.^110^ Increased neurogenesis in CMM in spring may therefore be related to auditory processing of male vocalizations, for example during mate selection, however, to our knowledge, no study has yet to examine this experimentally.

One possible direction may come from electrophysiological studies or studies using immediate‐early genes as proxies for neuronal activation. Tokarev et al.^28^ showed that new neurons recruited to the HVC‐RA circuit in adult zebra finches become activated (as measured by ZENK‐ and c‐Fos‐ir) as early as 3 weeks post‐mitosis, meaning increased recruitment to CMM in spring may support an increased demand on this region during the prebreeding and breeding period, perhaps to facilitate assessment of mate quality based on male vocal performance. Again, we must be cautious about drawing strong conclusions from these results with the relatively small number of females studied, however, understanding whether new neurons in CMM and NCM follow the same timeline of incorporation and activation in the same way as Tokarev et al.^28^ observed in HVC is not known and also warrants further investigation.

### 4.3 Seasonal relationships among physiological measures

Starlings had higher levels of plasma CORT in spring than in fall, which is consistent with the bulk of previous work in starlings^45^ and other free‐living species.^111^ However, HLR did not show a predictable change across seasons in European starlings, nor was plasma CORT correlated with HLR. Interpretation of HLRs may be complicated by the influence of factors such as immune function, hormones, and environmental factors.^47^ This may not be surprising if we consider that increased CORT levels, and possibly HLR levels, are not necessarily synonymous with increased stress, but instead are only two indicators in a repertoire of responses to various stressors and seasonal change.^112^

Although CORT (and stress more broadly) can inhibit neurogenesis,^44^, ^113^, ^114^, ^115^ we did not find a negative relationship between CORT and neurogenesis in any region or with any DCX‐ir cell type. Our study was designed to minimize stress associated with captivity, so perhaps this negative correlation reflects more accurately whether CORT affects neurogenesis in wild‐caught birds, although in captive song sparrows, exogenous CORT treatment did not affect neurogenesis.^22^ Ultimately, more research is likely needed to fully understand the relationship between CORT, stress, and neurogenesis in free‐living birds.

## 5 CONCLUSIONS

In this study, we describe seasonal changes in both physiology and in neurogenesis in vocal control and auditory perceptual regions of male and female European starlings. Physiologically, males captured in spring appeared to be in breeding condition and both males and females exhibited higher plasma CORT concentrations than those captured in fall, but CORT did not appear to inhibit neurogenesis. Our study, conducted with free‐living birds, is more likely to capture the variability of demands in naturalistic environments compared with laboratory studies. However, by not measuring the birds' behavior, and having a limited number of females captured in spring, our ability to speculate about the behavioral correlates of the neurobiological effects we show here is limited. Laboratory experiments in tandem with controlled field experiments will be useful in determining whether increased neurogenesis in female birds compared with males provides them with the readiness to shift between these behaviors throughout the year. Overall, the ability of songbirds to adjust their behavior in response to seasonal changes highlights the remarkable plasticity of their neuroendocrine system and underscores the importance of studying their neurobiology in a naturalistic context.

## AUTHOR CONTRIBUTIONS

**Sean D. T. Aitken:** Conceptualization; formal analysis; investigation; methodology; validation; visualization; writing – original draft; writing − review and editing. **Broderick. M. B. Parks:** Data curation; formal analysis; investigation; validation; visualization; writing – review and editing. **Marjorie Sollows:** Investigation. **Colleen A. Barber:** Methodology; resources; supervision. **Leslie S. Phillmore:** Conceptualization; funding acquisition; methodology; project administration; resources; supervision; visualization; writing – review and editing.

## FUNDING INFORMATION

This work was directly supported by a Discovery Grant (RGPIN‐2018‐04060) awarded to LSP by the Natural Sciences and Engineering Research Council of Canada (NSERC).

## CONFLICT OF INTEREST STATEMENT

The authors declare no conflicts of interest.

## PEER REVIEW

The peer review history for this article is available at https://www.webofscience.com/api/gateway/wos/peer-review/10.1111/jne.13455.

## Supporting information


**Figure S1.** Seasonal differences in stage of ovary development in female European starlings. Dots are individual data (*n* = 2 spring females; *n* = 4 fall females); dashed line indicates threshold for breeding condition (ovary stage ≥3).
**Figure S2.** Seasonal differences in counts of DCX‐ir round cells (per microscope field of view, FOV) in male, but not female, European starlings. Data presented are estimated marginal means ± bootstrapped 95% CIs generated from generalized linear mixed models. Blue circles represent data collected from birds captured in spring; orange triangles represent data collected from birds captured in fall. Double asterisk (**) indicates a significant sex × season interaction effect at *p* < .001.
**Figure S3.** Region‐specific seasonal differences in %DCX‐ir coverage (per microscope field of view, FOV) in European starlings. Data presented are estimated marginal means ± bootstrapped 95% CIs generated from general linear mixed models. Blue circles represent data collected from birds captured in spring; orange triangles represent data collected from birds captured in fall. Asterisk indicates a significant region × season interaction effect at *p* < .05.
**Figure S4.** Region‐specific sex differences in %DCX‐ir coverage (per microscope field of view, FOV) in European starlings. Data presented are estimated marginal means ± bootstrapped 95% CIs generated from general linear mixed models. Pink circles represent data collected from female birds; blue circles represent data collected from male birds. Asterisk (*) indicates a significant region × sex interaction effect at *p* < .05.
**Figure S5.** Heterophil:lymphocyte ratio (HLR) is significantly correlated with DCX‐ir multipolar cell counts (per microscope field of view, FOV) in auditory perceptual region NCM (caudomedial nidopallium) in European starlings (Pearson's *r* = −.61; *p* = .0025). Dots represent average DCX‐ir multipolar counts in NCM (across hemisphere and successive section; 1 dot = 1 bird; *n* = 14; 8 spring males, blue dots; 6 fall males, orange dots); solid line indicates regression line.
**Figure S6.** Testis volume is significantly correlated with DCX‐ir round cell counts (per microscope field of view, FOV) in vocal control nucleus HVC in male European starlings (Pearson's *r* = .70; *p* = .0036). Dots represent average DCX‐ir round cell counts in HVC (across hemisphere and successive section; 1 dot = 1 bird; *n* = 9; 6 spring males, blue dots; 3 fall males, orange dots); solid line indicates regression line.

## Data Availability

The data that support the findings of this study are available from the corresponding author upon reasonable request.
