# Supplementary material for: Seasonal patterns of neurogenesis in European starlings (Sturnus vulgaris) are region‐ and sex‐specific
Source: J Neuroendocrinol. 2024 Oct 16;37(6):e13455. doi: 10.1111/jne.13455 (PMC12145945; doi:10.1111/jne.13455)
Supplement: Supplementary file 1 — Figure S1. Seasonal differences in stage of ovary development in female European starlings. Dots are individual data (n = 2 spring females; n = 4 fall females); dashed line indicates threshold for breeding condition (ovary stage ≥3). Figure S2. Seasonal differences in counts of DCX‐ir round cells (per microscope field of view, FOV) in male, but not female, European starlings. Data presented are estimated marginal means ± bootstrapped 95% CIs generated from generalized linear mixed models. Blue circles represent data collected from birds captured in spring; orange triangles represent data collected from birds captured in fall. Double asterisk (**) indicates a significant sex × season interaction effect at p < .001. Figure S3. Region‐specific seasonal differences in %DCX‐ir coverage (per microscope field of view, FOV) in European starlings. Data presented are estimated marginal means ± bootstrapped 95% CIs generated from general linear mixed models. Blue circles represent data collected from birds captured in spring; orange triangles represent data collected from birds captured in fall. Asterisk indicates a significant region × season interaction effect at p < .05. Figure S4. Region‐specific sex differences in %DCX‐ir coverage (per microscope field of view, FOV) in European starlings. Data presented are estimated marginal means ± bootstrapped 95% CIs generated from general linear mixed models. Pink circles represent data collected from female birds; blue circles represent data collected from male birds. Asterisk (*) indicates a significant region × sex interaction effect at p < .05. Figure S5. Heterophil:lymphocyte ratio (HLR) is significantly correlated with DCX‐ir multipolar cell counts (per microscope field of view, FOV) in auditory perceptual region NCM (caudomedial nidopallium) in European starlings (Pearson's r = −.61; p = .0025). Dots represent average DCX‐ir multipolar counts in NCM (across hemisphere and successive section; 1 dot = 1 bird; n = 14; 8 spring ma [file JNE-37-e13455-s001.docx]

**Supplemental Materials for:**

**Seasonal patterns of neurogenesis in European starlings (*Sturnus vulgaris*) are region- and sex-specific**

Sean D. T. Aitken^1^, Broderick. M. B. Parks^1^, Marjorie Sollows^1^, Colleen A. Barber^2^ and Leslie S. Phillmore^1^*

*^1^Department of Psychology and Neuroscience, Dalhousie University, Halifax NS, Canada*

*^2^Department of Biology, Saint Mary’s University, Halifax NS, Canada*

***Corresponding author:** Leslie S. Phillmore ([Leslie.Phillmore@dal.ca](mailto:Leslie.Phillmore@dal.ca))

**Supplemental Figures**

**
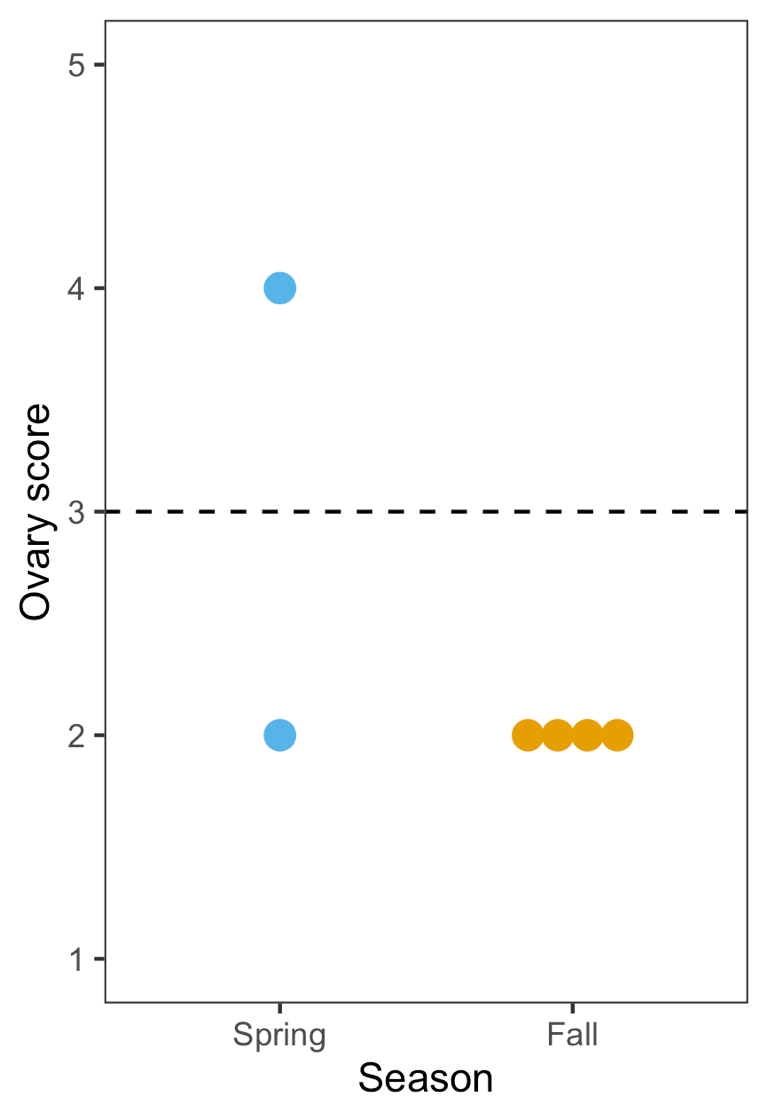
**

**Figure S1.** Seasonal differences in stage of ovary development in female European starlings.

Dots are individual data (*n*=2 spring females; *n*=4 fall females); dashed line indicates threshold for breeding condition (ovary stage ≥ 3).

**
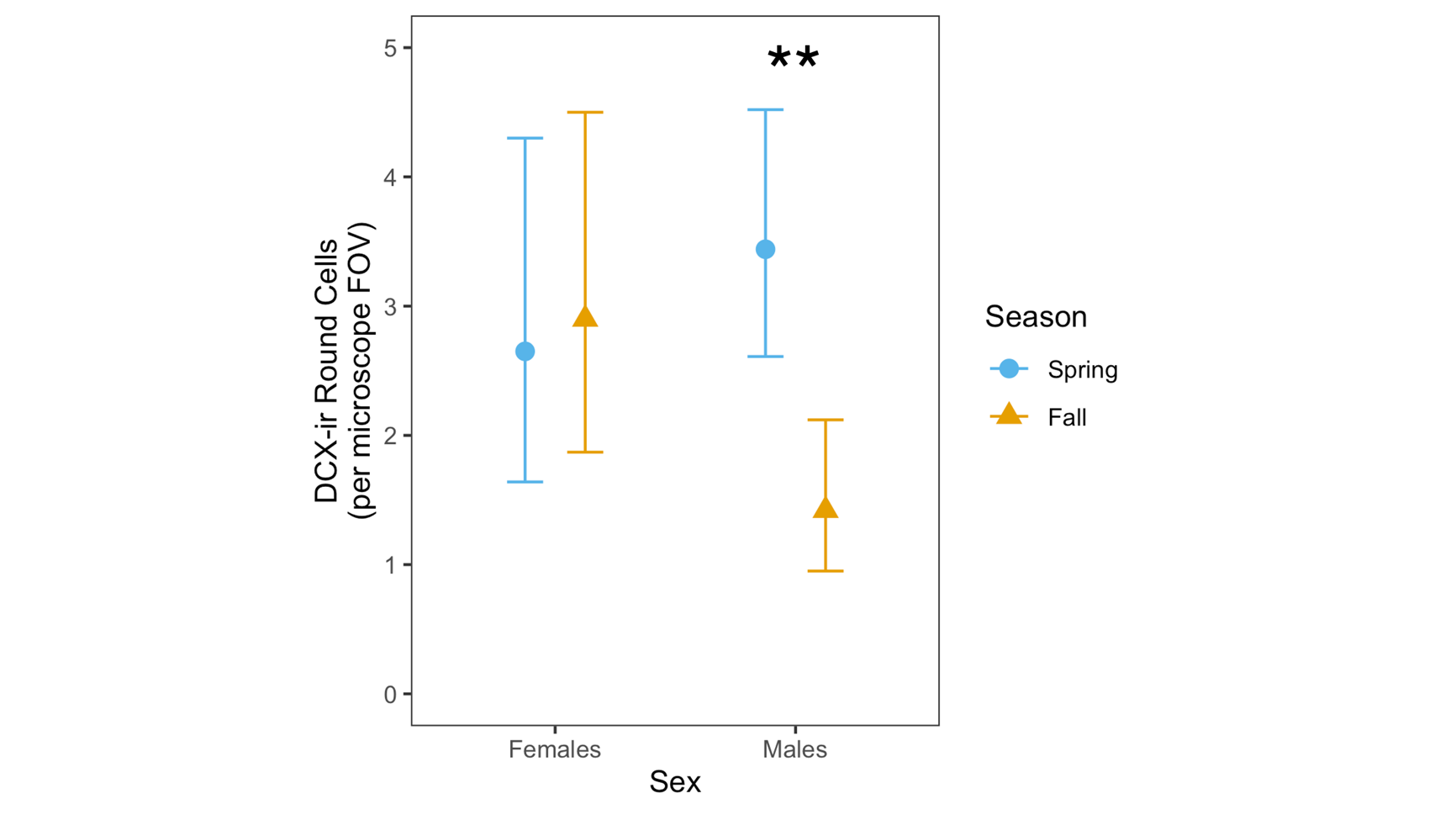
**

**Figure S2.** Seasonal differences in counts of DCX-ir round cells (per microscope field of view, FOV) in male, but not female, European starlings. Data presented are estimated marginal means ± bootstrapped 95% CIs generated from generalized linear mixed models. Blue circles represent data collected from birds captured in spring; orange triangles represent data collected from birds captured in fall. Double asterisk (**) indicates a significant sex × season interaction effect at *p*<0.001.

**
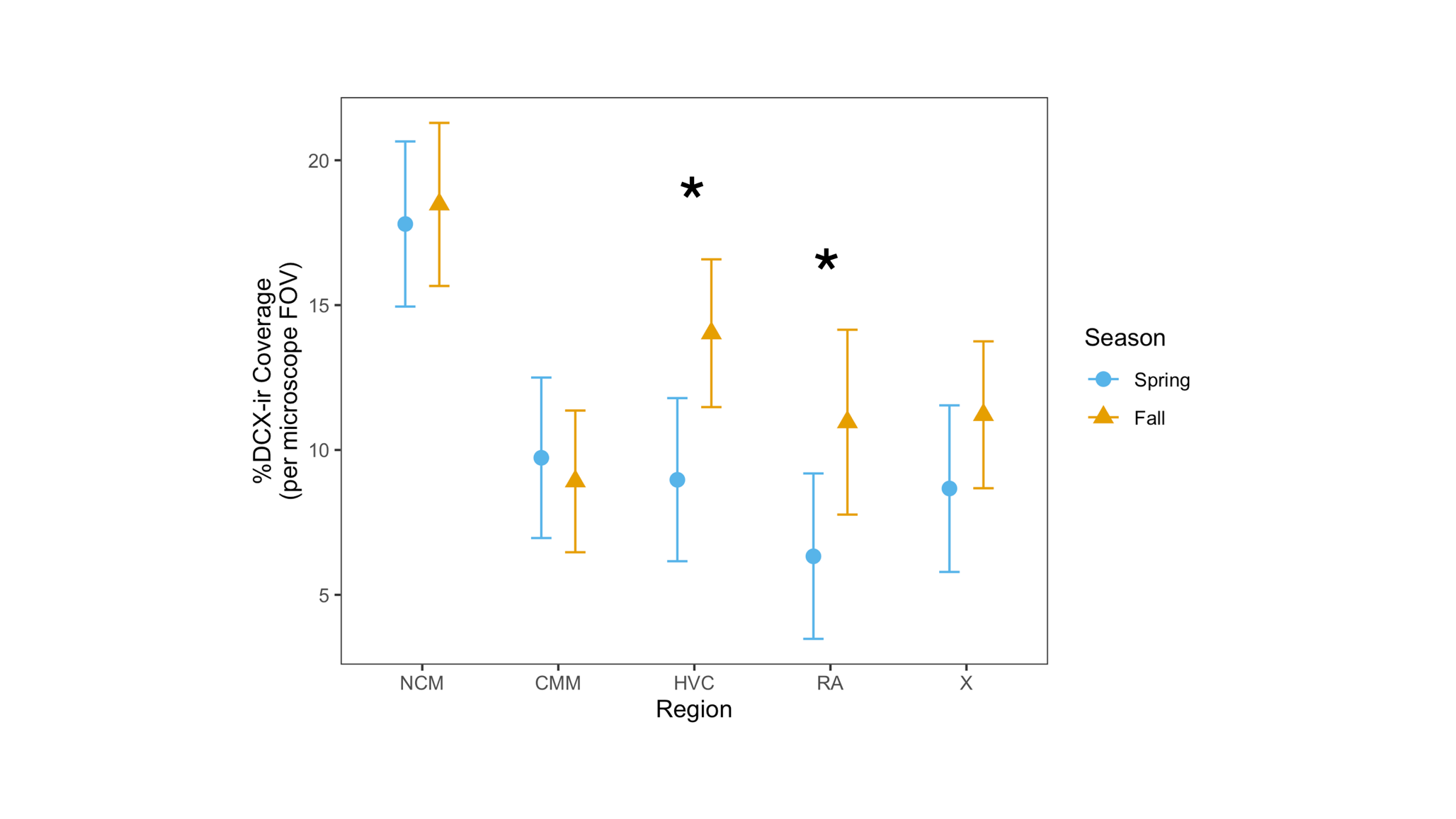
**

**Figure S3.** Region-specific seasonal differences in %DCX-ir coverage (per microscope field of view, FOV) in European starlings. Data presented are estimated marginal means ± bootstrapped 95% CIs generated from general linear mixed models. Blue circles represent data collected from birds captured in spring; orange triangles represent data collected from birds captured in fall. Asterisk indicates a significant region × season interaction effect at *p*<0.05.

**
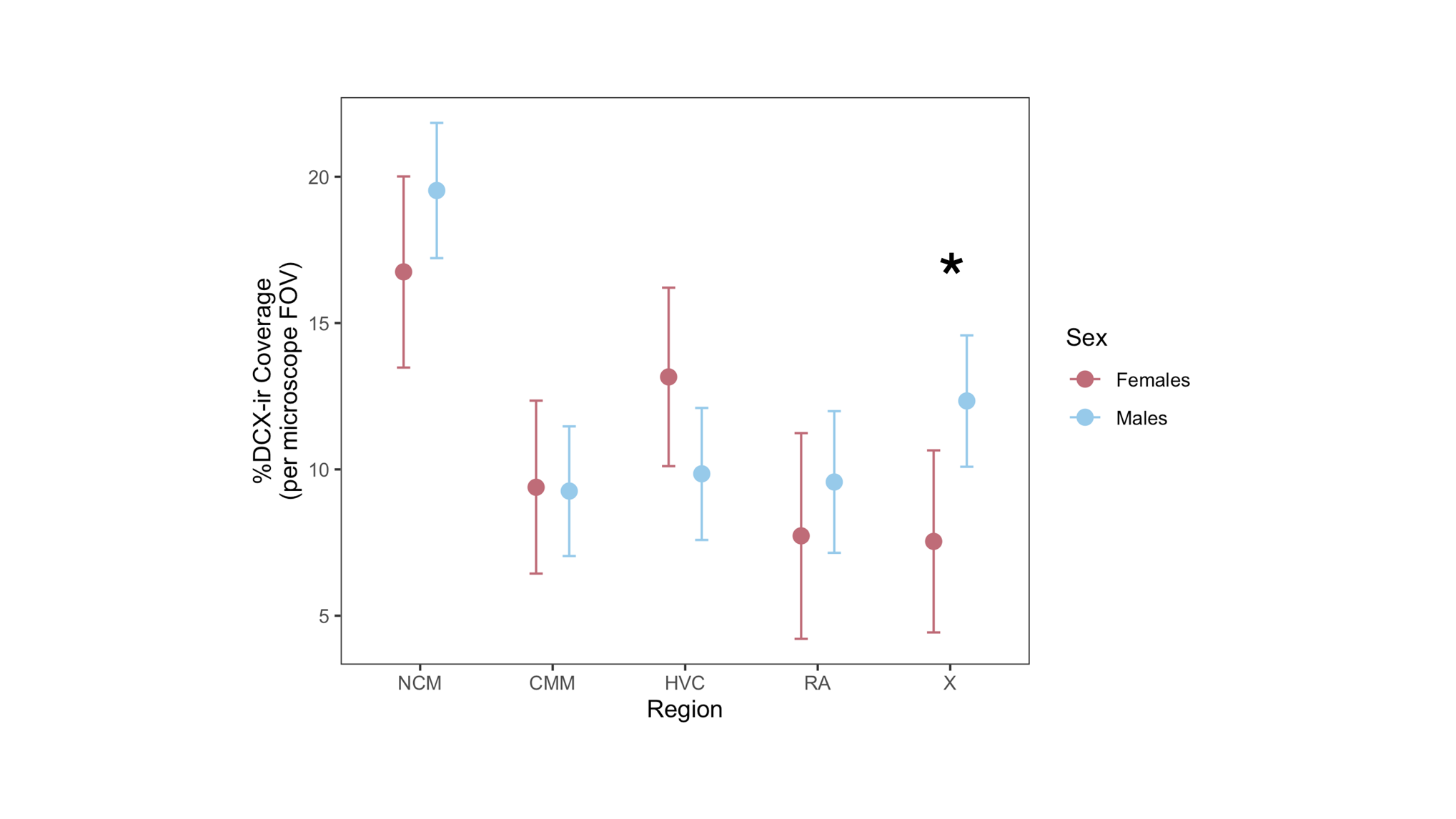
**

**Figure S4.** Region-specific sex differences in %DCX-ir coverage (per microscope field of view, FOV) in European starlings. Data presented are estimated marginal means ± bootstrapped 95% CIs generated from general linear mixed models. Pink circles represent data collected from female birds; blue circles represent data collected from male birds. Asterisk (*) indicates a significant region × sex interaction effect at *p*<0.05.

**
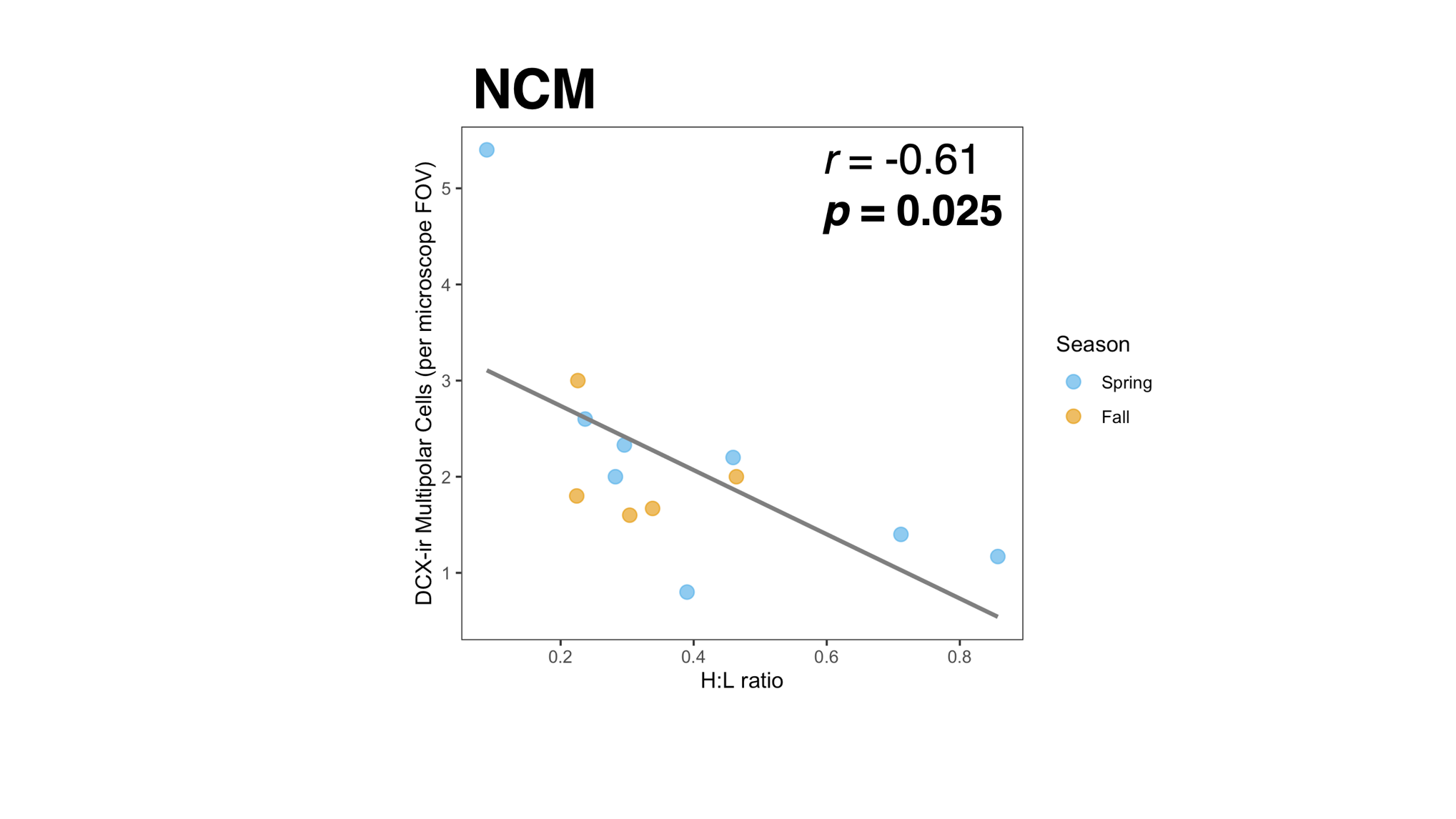
**

**Figure S5.** Heterophil:leukocyte ratio (H:L ratio) is significantly correlated with DCX-ir multipolar cell counts (per microscope field of view, FOV) in auditory perceptual region NCM (caudomedial nidopallium) in European starlings (Pearson’s *r* = -0.61; *p* = 0.0025). Dots represent average DCX-ir multipolar counts in NCM (across hemisphere and successive section; 1 dot = 1 bird; *n*=14; 8 spring males, blue dots; 6 fall males, orange dots); solid line indicates regression line.

**
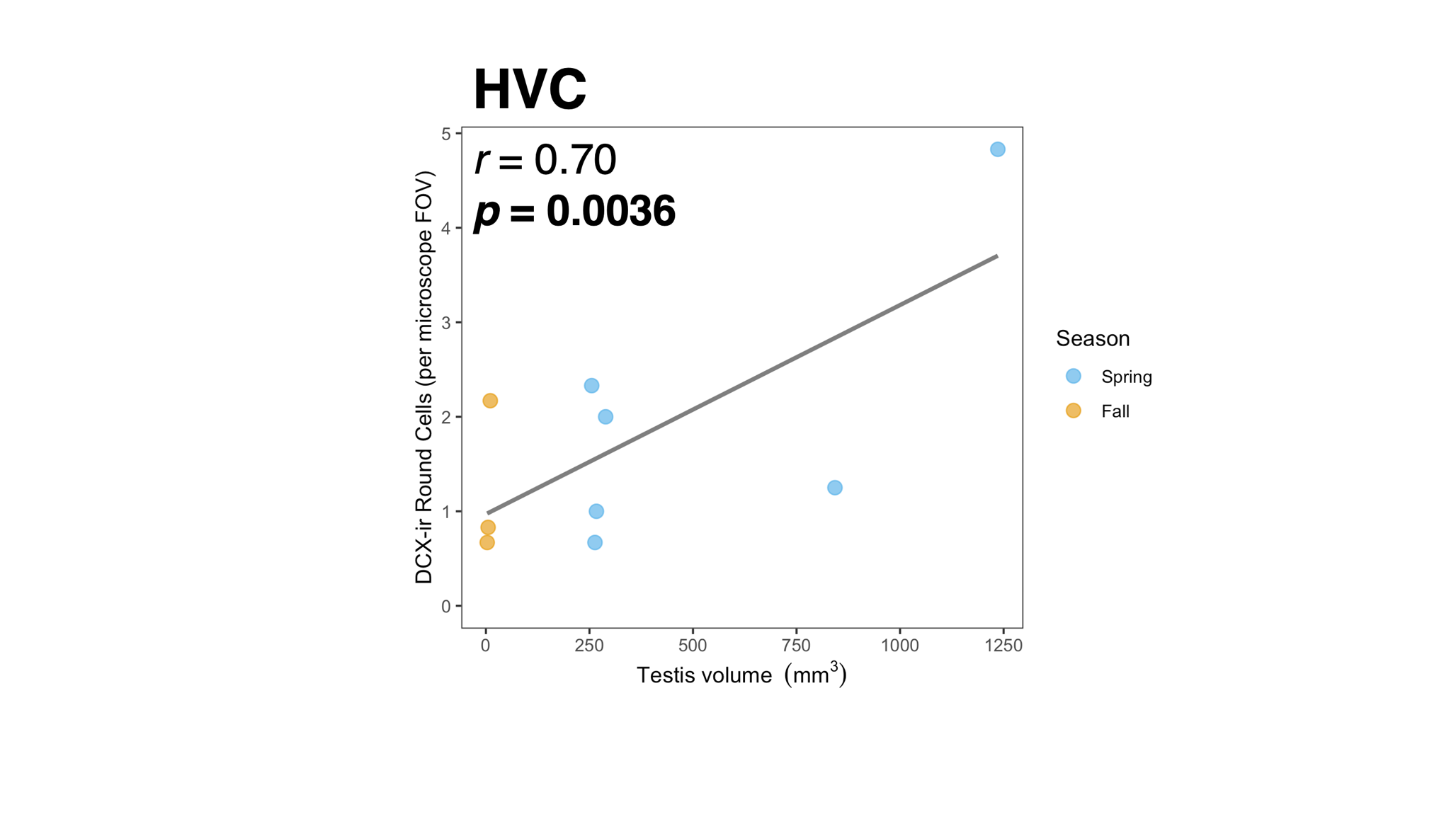
**

**Figure S6.** Testis volume is significantly correlated with DCX-ir round cell counts (per microscope field of view, FOV) in vocal control nucleus HVC in male European starlings (Pearson’s *r* = 0.70; *p* = 0.0036). Dots represent average DCX-ir round cell counts in HVC (across hemisphere and successive section; 1 dot = 1 bird; *n*=9; 6 spring males, blue dots; 3 fall males, orange dots); solid line indicates regression line.
